# CCR5 as a Therapeutic Target in HIV Disease: From CRISPR/Cas9 Gene Editing to Maraviroc-Mediated Inhibition

**DOI:** 10.3390/v18090972

**Published:** 2026-09-03

**Authors:** Uzair Iqbal, Khadija Khalid, Mohamed Shaltout, Yunus Yukselten, Richard E. Sutton

**Affiliations:** Section of Infectious Diseases, Department of Medicine, Yale School of Medicine, New Haven, CT 06520, USA; uzair.iqbal@yale.edu (U.I.); khadija.khalid@yale.edu (K.K.); yunus.yukselten@yale.edu (Y.Y.)

**Keywords:** CCR5, Crispr/Cas9, maraviroc, HIV cure, gene editing

## Abstract

The C-C chemokine receptor type 5 (CCR5) is the principal co-receptor for R5-tropic HIV-1 and remains one of the most promising therapeutic targets in the pursuit of an HIV cure. The discovery that individuals carrying the naturally occurring CCR5Δ32 mutation exhibit marked resistance to HIV infection established the foundation for both genetic and pharmacological approaches to CCR5 inhibition. This review summarizes recent advances in CCR5-targeted therapies with a focus on CRISPR/Cas9-mediated gene editing and maraviroc-mediated receptor blockade. We discuss the molecular mechanisms, preclinical evidence and emerging clinical data supporting CRISPR-based CCR5 disruption, including multiplex editing strategies designed to overcome viral tropism switching. We also examine the evolving role of maraviroc beyond viral entry inhibition, highlighting its immunomodulatory effects, potential latency-reversing activity and applications in graft-versus-host disease and cancer. Together, these complementary strategies underscore the potential of CCR5-targeted interventions as integral components of future combination therapies aimed at achieving durable HIV remission or functional cure.

## 1. Introduction:

C-C chemokine receptor type 5 (CCR5) is a G protein-coupled receptor expressed on CD4+ T lymphocytes, monocytes, macrophages and NK cells that serves as the principal co-receptor for R5-tropic HIV-1 strains, facilitating viral entry into host cells [1]. The discovery that individuals homozygous for a naturally occurring 32-base-pair deletion in the CCR5 gene (CCR5Δ32) exhibit near-complete resistance to HIV-1 infection provided the foundational rationale for targeting this receptor therapeutically [2]. Clinical proof of concept was established through the landmark “Berlin” and “London” patients, both of whom achieved long-term HIV-1 remission following allogeneic hematopoietic stem cell transplantation from CCR5Δ32/Δ32 donors [3]. However, the extreme rarity of HLA-matched CCR5Δ32 homozygous donors, with a prevalence of approximately 1% in Caucasian populations, combined with the morbidity and mortality of allogeneic transplantation, renders this approach impractical for the vast majority of people living with HIV [4].

This limitation has catalyzed the development of two complementary therapeutic strategies targeting CCR5. First, CRISPR/Cas9-based gene editing has emerged as a powerful tool for artificially disrupting CCR5 in autologous or allogeneic hematopoietic stem and progenitor cells (HSPCs) [1]. Preclinical studies have demonstrated that CRISPR-mediated CCR5 ablation confers HIV-1 resistance in humanized mouse models, with dual CRISPR strategies targeting both CCR5 and integrated proviral DNA, achieving viral elimination in 58% of infected animals [5]. The first in-human clinical application reported in the New England Journal of Medicine demonstrated that CRISPR-edited CCR5-ablated HSPCs could be safely transplanted with long-term engraftment, although the editing efficiency of approximately 5% was insufficient for HIV cure [6]. More recently, in vivo base-editing strategies have achieved approximately 50% CCR5 editing in bone marrow cells, conferring 12-fold-lower HIV plasma titers in humanized mice [7]. Simultaneously, novel multiplex editing approaches, e.g., combining CCR5 knockout with anti-CXCR4 strategies, have addressed the critical vulnerability of viral tropism switching, achieving up to 2000-fold reduction in X4-tropic replication [8].

Second, maraviroc, the only FDA-approved small-molecule CCR5 antagonist, continues to reveal multifaceted therapeutic dimensions beyond its canonical role as an entry inhibitor [9]. Recent evidence has repositioned maraviroc as a potential latency-reversing agent, capable of reactivating latent HIV-1 through NF-κB activation via CCR5 engagement, with potency comparable to established latency-reversing agents such as bryostatin-1 [10]. A 2025 longitudinal study further demonstrated that initiating antiretroviral therapy with maraviroc resulted in a 4- to 7-fold-greater reduction in integrated HIV-DNA reservoir compared to non-maraviroc regimens, supporting its role in “kick and kill” cure strategies [11]. Beyond HIV, maraviroc-mediated CCR5 blockade has shown therapeutic promise in graft-versus-host disease prophylaxis and cancer, where the CCL5/CCR5 axis governs immunosuppressive cell recruitment and tumor progression [12].

This review comprehensively examines CCR5 as a dual therapeutic target, synthesizing the current evidence on CRISPR/Cas9-mediated gene editing, from mechanistic advances and preclinical validation to first-in-human clinical translation alongside the evolving pharmacological landscape of maraviroc-mediated CCR5 inhibition, charting a path toward scalable, combinatorial strategies for achieving a functional HIV cure.

## 2. Role of CRISPR/Cas9 in CCR5 Gene Regulation

**Figure 1:**
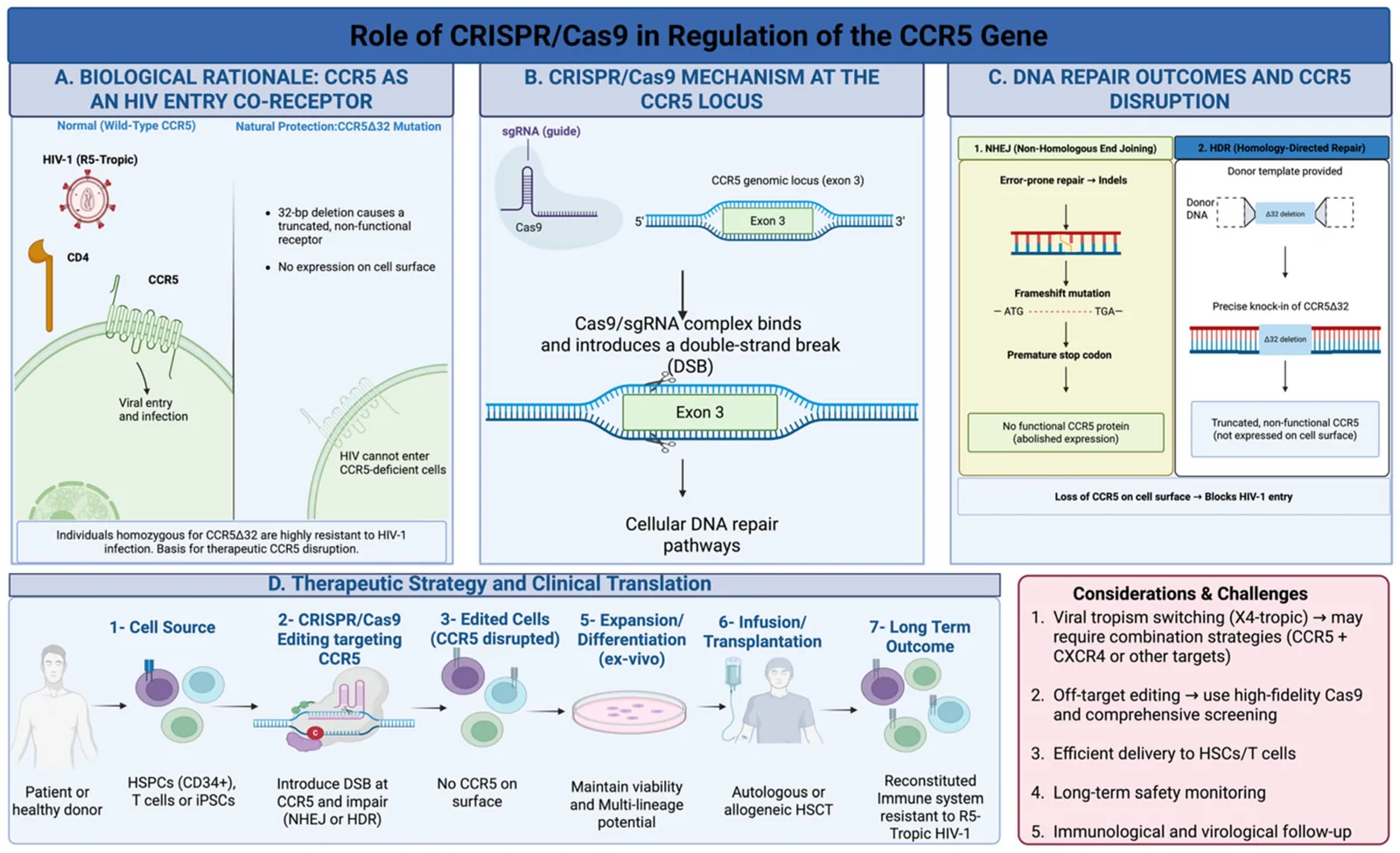
CRISPR/Cas9-mediated disruption of CCR5 for HIV therapy.

The C-C chemokine receptor type 5 (CCR5) is a G protein-coupled receptor expressed on the surface of CD4+ T lymphocytes, monocytes, dendritic cells, macrophages and NK cells. It serves as the principal co-receptor for R5-tropic HIV-1 strains, facilitating viral entry into host cells [1]. The discovery that individuals homozygous for a naturally occurring 32-base-pair deletion in the CCR5 gene (CCR5Δ32) exhibit almost complete resistance to HIV-1 infection provided the foundational rationale for targeting this gene therapeutically [2]. This natural mutation produces a truncated, non-functional receptor that cannot be expressed on the cell surface, so R5-tropic HIV-1 attachment and entry is unable to occur. [13] The prevalence of the CCR5Δ32 allele is approximately 10% in European populations and homozygosity occurs in roughly 1% of Caucasians [4]. The clinical proof-of-concept was established through the “Berlin patient” and “London patient,” both of whom achieved long-term HIV-1 remission following allogeneic hematopoietic stem cell transplantation (HSCT) from CCR5Δ32 homozygous donors [1]. However, the extreme rarity of HLA-matched CCR5Δ32 homozygous donors, combined with the morbidity and mortality associated with allogeneic HSCT, renders this approach impractical for the vast majority of people living with HIV [4]. This limitation has driven the development of gene-editing strategies, particularly CRISPR/Cas9, to artificially disrupt CCR5 in autologous or allogeneic cells (shown in Figure 1).

### 2.1. Mechanism of CRISPR/Cas9-Mediated CCR5 Disruption

The CRISPR/Cas9 system employs a single guide RNA (sgRNA) to direct the Cas9 endonuclease to a specific genomic locus, where it introduces a double-strand break (DSB). The cell’s endogenous repair machinery then processes this DSB by one of two primary pathways: non-homologous end joining (NHEJ) or homology-directed repair (HDR) [14]. NHEJ is error prone and frequently introduces insertions or deletions at the cleavage site, resulting in frameshift mutations that abolish CCR5 protein expression [14]. Unlike NHEJ, HDR uses a supplied donor template to mediate highly precise DNA repair, enabling the seamless recreation of the protective CCR5Δ32 deletion [15]. Ye et al. demonstrated that CRISPR/Cas9 combined with piggyBac transposon technology could generate iPSCs (induced pleuripotent stem cells) carrying the exact CCR5Δ32 mutation with biallelic targeting efficiency of up to 33% and that monocytes/macrophages differentiated from these edited iPSCs were resistant to HIV-1 challenge [15]. Scheller et al. further advanced this approach by developing a biallelic selectable knock-in strategy using CRISPR/Cas9-mediated HDR, enabling efficient selection of cells with frameshift mutations in both CCR5 alleles, which displayed potent inhibition of HIV-1 infection [16]. Multiple Cas9 variants have been employed for CCR5 editing. The standard Streptococcus pyogenes Cas9 (SpCas9) has been the most widely used, but the smaller Staphylococcus aureus Cas9 (SaCas9) offers advantages in cell delivery due to its reduced size and has been shown to efficiently disrupt CCR5 in both primary CD4+ T cells and CD34+ hematopoietic stem/progenitor cells (HSPCs) without impairing differentiation capacity [17]. Additionally, a high-fidelity Cas9 variant (HiFi Cas9, bearing the R691A mutation) has been developed to maintain high on-target activity while substantially reducing off-target editing and has demonstrated robust gene targeting at the CCR5 locus in HSPCs delivered as ribonucleoprotein complexes [18].

### 2.2. Preclinical Evidence in Cell and Animal Models

Preclinical studies have consistently demonstrated that CRISPR/Cas9-mediated CCR5 disruption confers HIV-1 resistance across diverse cellular and animal models (Table 1). Xu et al. established a CRISPR/Cas9 system in human CD34+ HSPCs that achieved efficient CCR5 ablation, with the disruption efficiency remaining robust through secondary transplantation in immunodeficient mice. Humanized mice engrafted with CCR5-edited cells showed significant reduction in viral titers and selective enrichment of CD4+ T cells upon HIV-1 challenge [19]. Similarly, Xiao et al. demonstrated that SaCas9-mediated CCR5 disruption in primary CD4+ T cells led to selective survival and enrichment of edited cells in humanized mice challenged with R5-tropic HIV-1 [17]. A landmark preclinical study by Dash et al. employed a dual CRISPR/Cas9 strategy targeting both CCR5 and integrated HIV-1 proviral DNA in humanized mice receiving antiretroviral therapy [5]. This combined approach achieved elimination of replication-competent virus in 58% of infected animals, with no detectable HIV-1 in blood, spleen, lung, kidney, liver, gut, bone marrow, or brain. Viral outgrowth assays and adoptive transfer experiments confirmed viral elimination and no off-target toxicities were observed. Importantly, the dual CRISPR therapy showed statistically significant improvements over single-target approaches [5]. More recently, Anderson et al. presented an in vivo base-editing strategy using helper-dependent adenoviral vectors expressing base editors targeting CCR5 in HSCs. In a humanized mouse model, in vivo transduction achieved approximately 50% base editing at the CCR5 target site in bone marrow mononuclear cells, conferring approximately 12-fold-lower HIV plasma titers compared to controls after HIV challenge, with no significant off-target editing [7].

### 2.3. Clinical Translation: First-in-Human Evidence

The first clinical application of CRISPR/Cas9-mediated CCR5 editing was reported by Xu et al. in the New England Journal of Medicine. A patient with both HIV-1 infection and acute lymphoblastic leukemia received allogeneic HSCT with CRISPR-edited, CCR5-ablated donor HSPCs [6]. The leukemia achieved complete remission with full donor chimerism and donor cells carrying the ablated CCR5 persisted for more than 19 months without gene editing-related adverse events that include unintended off-target or on-target genomic alterations, chromosomal rearrangements and potential long-term effects on the safety and function of edited cells. During a brief period of antiretroviral therapy interruption, the percentage of CD4+ cells with CCR5 ablation increased modestly, suggesting a selective advantage for edited cells in the presence of viral pressure. However, the overall CCR5 disruption efficiency in lymphocytes was only approximately 5%, which was insufficient to achieve HIV-1 cure [6]. This study nonetheless provided critical proof of principle that CRISPR-edited HSPCs can be safely transplanted and engrafted long-term in humans, with CCR5 disruption maintained across multiple hematopoietic lineages.

### 2.4. Addressing the Limitation of Viral Tropism Switching

A significant limitation of CCR5-only disruption is that it does not protect against CXCR4-tropic (X4-tropic) HIV-1 strains, which can emerge under selective pressure. Several combined strategies have been developed to address this vulnerability. Khamaikawin et al. combined CRISPR/Cas9-mediated CCR5 knockout with expression of C46, a membrane-anchored HIV-1 fusion inhibitor, achieving resistance to both R5- and X4-tropic HIV-1 in cell line models, with the combined approach proving superior to either strategy alone [4]. Dudek et al. developed a simultaneous knock out–knock in genome-editing strategy in HSPCs using Cas9/AAV6 that achieved complete loss of R5-tropic replication and up to a 2000-fold decrease in X4-tropic replication without disrupting the CXCR4 locus itself, while maintaining multi-lineage repopulation capacity. [8] Li et al. demonstrated that simultaneous CRISPR-mediated disruption of both CCR5 and CXCR4 in T cells conferred broad resistance to R5-, X4- and dual-tropic HIV-1 strains, though CXCR4 disruption raised concerns about impaired bone marrow engraftment in CD4+ T cells [20].

An important cell-type-specific safety consideration arises with CXCR4 co-targeting strategies. While CRISPR-mediated CXCR4 disruption can be applied to mature T cells and macrophages to confer resistance to X4-tropic HIV-1 strains, this approach cannot be safely applied to HSPCs or bone marrow-resident cells [21]. The CXCL12/CXCR4 signaling axis is indispensable for the retention and maintenance of HSPCs within the bone marrow niche [22]. CXCR4 is the only chemokine receptor through which HSPCs migrate toward SDF-1 (CXCL12) gradients in the bone marrow microenvironment and disruption of this axis in HSPCs results in severely impaired bone marrow engraftment, reduced HSPC quiescence and compromised hematopoietic reconstitution [23]. Mice with induced CXCR4 deletion in HSCs show marked reduction in stem cell pool maintenance, increased oxidative stress and defective multi-lineage differentiation [24]. Therefore, dual CCR5/CXCR4 gene-editing strategies must be restricted to differentiated immune effector cells such as T cells and macrophages, while preserving CXCR4 expression in the stem cell compartment to ensure proper bone marrow homing and long-term hematopoietic function.

### 2.5. Safety Considerations and Off-Target Effects

Safety remains a central concern for clinical translation of CRISPR/Cas9-based CCR5 editing. Off-target mutagenesis, where Cas9 cleaves unintended genomic sites, poses risks of insertional oncogenesis or disruption of essential genes. Mandal et al. examined on- and off-target mutations via target capture sequencing in HSPCs edited at the CCR5 locus and observed low levels of off-target mutagenesis at only one site [25]. The development of high-fidelity Cas9 variants has further mitigated this risk [18]. In the first-in-human clinical study, no gene editing-related adverse events were detected over 19 months of follow-up. [6] Additionally, it should be noted that homozygous CCR5Δ32 carriers, while resistant to HIV-1, display increased susceptibility to West Nile virus encephalomyelitis, highlighting that CCR5 disruption is not without potential immunological or virological consequences [2]. Beyond West Nile virus susceptibility, evidence suggests that CCR5∆32 carries disadvantages in other infectious diseases. In the 2009 H1N1 pandemic, ∆32 carriers showed significantly higher influenza mortality than wild-type individuals (17.4% vs. 4.7%), implicating CCR5 deficiency in fatal outcomes, although several cohorts found no such association [26,27,28]. In HPV-infected individuals, the rare ∆32/∆32 genotype has been linked to increased infection risk [29]. Most strikingly, Wei and Nielsen reported that ∆32 homozygotes in the UK Biobank population had a greater-than-20% increase in all-cause mortality and an almost 2-year reduction in lifespan [30].

The integration of CCR5-targeted gene editing with immune-based interventions and multiplex editing strategies targeting CCR5, CXCR4 and HIV long terminal repeat (LTR) loci simultaneously represents a promising frontier. However, key challenges including potential immune responses against gene-editing components or edited cells, viral tropism switching, delivery efficiency, economic feasibility and long-term safety monitoring must be resolved before these approaches can be broadly implemented [1].

## 3. CCR5 as the Therapeutic Target of Maraviroc

**Figure 2:**
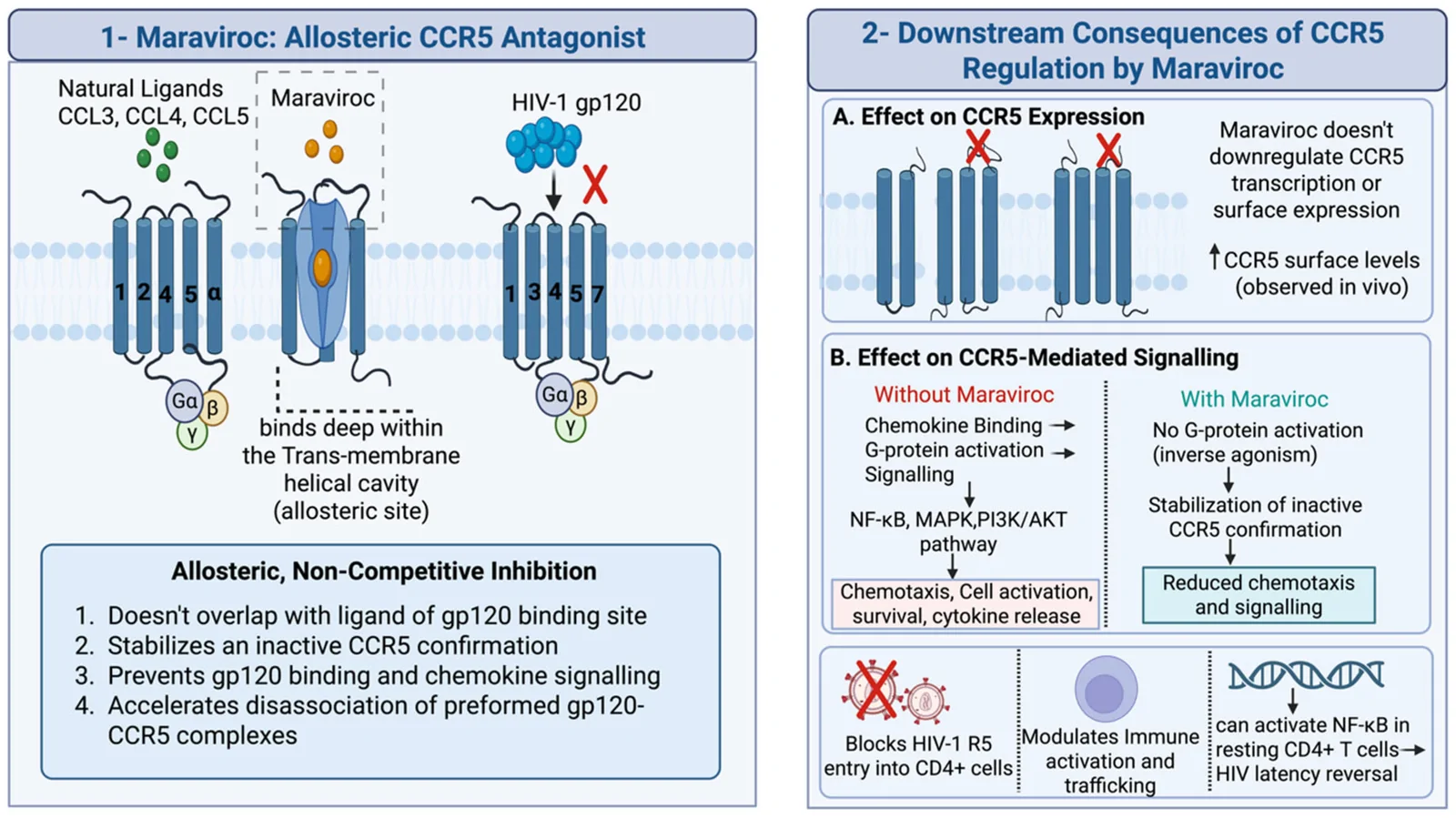
Mechanism of maraviroc-mediated CCR5 inhibition.

Maraviroc (marketed as Selzentry/Celsentri) is the first and only FDA-approved small-molecule CCR5 antagonist. It selectively binds to the transmembrane helical cavity of CCR5, inducing a conformational change that prevents the interaction between the HIV-1 envelope glycoprotein gp120 and the CCR5 coreceptor, thereby blocking viral entry into CD4+ T cells [31]. Importantly, maraviroc operates through an allosteric noncompetitive mechanism. It does not bind to the same extracellular sites as gp120 or natural chemokine ligands but instead inserts deeply into the transmembrane domain, stabilizing a CCR5 conformation that is unfavorable for both gp120 and chemokine engagement [32]. Garcia-Perez et al. demonstrated that maraviroc functions as a weak inverse agonist of CCR5, stabilizing receptor conformations with impaired G protein coupling, and that it accelerates the dissociation of preformed gp120-CCR5 complexes [32]. Critically, early pharmacological characterization confirmed that maraviroc does not alter CCR5 cell surface expression levels or trigger receptor internalization, distinguishing it from chemokine agonists such as PSC-RANTES, which inhibit HIV partly through CCR5 receptor downregulation [33]. The action of maraviroc on the CCR5 gene is shown in Figure 2.

### 3.1. Effects of Maraviroc on CCR5 Expression and Immune Parameters In Vivo

Although maraviroc does not directly downregulate CCR5 transcription or its surface density, its administration in clinical settings produces measurable immunological consequences related to CCR5 regulation. In a 48-week placebo-controlled randomized trial of maraviroc intensification in HIV-1-infected patients with suboptimal CD4+ recovery, van Lelyveld et al. found that maraviroc increased the percentage of CCR5-expressing CD4+ and CD8+ T cells and elevated plasma levels of the CCR5 ligand MIP-1β (CCL4) while simultaneously reducing ex vivo T-cell apoptosis [34]. Similarly, Hunt et al. reported in a randomized trial that maraviroc intensification led to a 2.4-fold increase in plasma MIP-1β levels and unexpectedly increased T-cell activation markers (CD38+HLA-DR+) on both CD4+ and CD8+ T cells in peripheral blood and rectal tissue [35]. These paradoxical findings suggest that CCR5 blockade by maraviroc may redirect endogenous CCR5 ligands toward alternative chemokine receptors (such as CCR1 or CCR3), triggering compensatory immune activation pathways [35]. These observations highlight that pharmacological CCR5 antagonism does not simply silence CCR5-mediated signaling but rather reshapes the broader chemokine network.

The absence of CCR5 downregulation is critical because maraviroc’s therapeutic logic in cure regimens rests on removing the receptor that R5-tropic HIV requires for entry, yet the drug leaves CCR5 transcription and surface density intact and even increases the percentage of CCR5-expressing CD4+ and CD8+ T cells while raising MIP-1β (CCL4) [34,35]. This means susceptible target cells persist rather than being eliminated and displaced ligands can activate alternative receptors (e.g., CCR1/CCR3), driving paradoxical immune activation [35]. For “block-and-lock” or reservoir-reduction goals this is counterproductive; instead maraviroc’s real cure relevance lies in NF-κB-mediated latency reversal, though prolonged dosing failed to shrink the reservoir or prevent rebound [10,36].

### 3.2. Maraviroc as a Latency-Reversing Agent Through CCR5-Mediated NF-κB Activation

A particularly novel dimension of maraviroc’s interaction with CCR5 regulation involves its capacity to reactivate latent HIV-1. Madrid-Elena et al. conducted a phase II clinical study in which maraviroc was administered for 10 days to 20 HIV-1-infected individuals on suppressive antiretroviral therapy. The study demonstrated that maraviroc increased HIV-1 unspliced RNA transcripts in resting CD4+ T cells, accompanied by activation of the transcription factor NF-κB [10]. Mechanistic experiments in CCR5-expressing HeLa cells confirmed that maraviroc induces NF-κB activity specifically through CCR5 binding, as co-administration of the CCR5 inhibitor TAK779 blocked this effect [10]. López-Huertas et al. corroborated these findings in primary latently infected resting CD4+ T cells, showing that maraviroc at clinically relevant concentrations reactivated both X4- and R5-tropic latent HIV-1 with potency comparable to the PKC agonist bryostatin-1 [37]. These data position maraviroc as a potential latency-reversing agent (LRA), capable of modulating CCR5-dependent intracellular signaling cascades, specifically the NF-κB pathway, to drive transcription from the HIV-1 long terminal repeat (LTR). This represents a paradigm shift from viewing maraviroc solely as an entry inhibitor to recognizing its role in regulating downstream gene expression through CCR5 engagement.

### 3.3. Clinical Efficacy Data of Maraviroc

Maraviroc’s pivotal efficacy was established in the treatment-experienced setting by the twin phase 3 MOTIVATE 1 and MOTIVATE 2 trials, which enrolled patients with R5-tropic HIV-1, triple-class experience or resistance and viral loads >5000 copies/mL. Added to optimized background therapy (OBT), maraviroc produced substantially greater viral suppression than the placebo: in the pooled FDA analysis, 56% versus 22% achieved <400 copies/mL and 46% versus 17% achieved <50 copies/mL at week 48, with a mean HIV-1 RNA reduction of −1.84 versus −0.78 log_10_ copies/mL [38]. CD4 gains were also greater with maraviroc (increases of ~113–128 vs. 54–69 cells/mm^3^) [38]. In the treatment-naive setting, the MERIT trial compared twice-daily maraviroc with efavirenz, each with zidovudine/lamivudine. Maraviroc met noninferiority for <400 copies/mL (70.6% vs. 73.1%) but narrowly missed it for <50 copies/mL (65.3% vs. 69.3%) in the primary 48-week analysis [39]. A post hoc reanalysis excluding the ~15% of patients found to harbor non-R5 virus by the enhanced-sensitivity Trofile assay restored noninferiority on both endpoints [9]. At week 96 using the enhanced assay, virologic response rates were essentially identical (64% vs. 64% for <400 copies/mL) and 5-year follow-up confirmed durable, comparable suppression (50.8% vs. 45.9% < 50 copies/mL) with a numerically greater CD4 rise (293 vs. 271 cells/mm^3^) [40]. A retrospective deep-sequencing reanalysis of MERIT similarly indicated that maraviroc would have been noninferior had a more sensitive screening method been used [41].

As a switch strategy, the randomized MARCH study showed that replacing a ritonavir-boosted PI with maraviroc while maintaining a 2-NRTI backbone was noninferior for maintaining suppression, whereas an NRTI-sparing maraviroc + PI/r regimen was inferior [42]. Small nucleoside-sparing dual-therapy studies (VEMAN, maraviroc + darunavir/ritonavir pilot, maraviroc + atazanavir/ritonavir) achieved high suppression rates in selected R5 naive patients but were underpowered and a meta-analysis of dual-drug regimens found the inferiority signal of dual therapy was largely driven by maraviroc-containing arms [43,44].

### 3.4. Safety and Adverse Effects

Maraviroc is generally well tolerated with a placebo-like adverse event profile in controlled trials. The most common events exceeding the placebo in treatment-experienced adults were upper respiratory tract infections, cough, pyrexia, rash and dizziness. Discontinuation for adverse events was 5% in both maraviroc and placebo arms. Notably, in both MERIT and its 5-year extension, fewer patients discontinued maraviroc than efavirenz for toxicity (10.6% vs. 21.3% at 5 years) [40].

The most clinically important safety concerns are captured in warnings, e.g., hepatotoxicity, sometimes preceded by systemic allergic features (pruritic rash, eosinophilia, elevated IgE), severe skin problems and hypersensitivity reactions including Stevens–Johnson syndrome, toxic epidermal necrolysis and DRESS [45]. Hepatic laboratory parameters should be checked before initiation and if rash or signs of hepatitis develop [45]. Despite these labeled warnings, the aggregate development-program hepatic analysis (2350 recipients) found no dose relationship, no signal of excess hepatic enzyme abnormalities versus comparators through week 96 and no cases meeting Hy’s Law [46]. A dedicated 144-week randomized trial in patients coinfected with HBV and/or HCV confirmed no increased hepatotoxicity and even suggested possible reductions in fibrosis markers/elastography, consistent with MOTIVATE subgroup analyses [47]. A cardiovascular signal (more myocardial ischemia/infarction in treatment-experienced recipients) also warrants monitoring [45].

### 3.5. Drug Accessibility and Tropism Testing

Maraviroc requires confirmation of exclusively CCR5-tropic virus before initiation, because CCR5 antagonists are ineffective against X4- or dual/mixed (D/M)-tropic virus and their use in such patients risks virologic failure and resistance to companion drugs [48]. Tropism can shift from R5 to X4/D/M with advancing immunodeficiency (particularly CD4 < 100 cells/µL), so IDSA recommends repeat testing before initiating a CCR5 antagonist even if prior testing was R5, unless a prior result was X4/D/M [48].

Phenotypic testing (Trofile) is the DHHS-preferred method, requiring HIV RNA ≥ 1000 copies/mL. The enhanced-sensitivity version detects CXCR4-utilizing clones down to ~0.3% of the population and replaced the original, less sensitive assay implicated in early failures [49]. Genotypic V3-loop assays interpreted with algorithms such as geno2pheno show high specificity (~90%) but only modest sensitivity (~50–75%) for CXCR4 virus [19]. Nonetheless, in the prospective phase 3 MODERN study, genotypic and phenotypic screening produced comparable maraviroc response rates and positive predictive values while the ANRS GenoTropism study supported the clinical utility of genotyping [49,50]. European guidelines accept genotyping as an equivalent option, whereas DHHS preferentially recommends phenotyping [49].

Genotypic algorithms overestimate CXCR4 usage in non-B subtypes (e.g., CRF01*AE*, *CRF02*AG), an important limitation in diverse populations [51]. For virologically suppressed patients, proviral DNA assays are available but of incompletely defined clinical utility [51]. Dosing is complicated by CYP3A/P-gp metabolism, requiring 150 mg (with potent CYP3A inhibitors), 300 mg (noninteracting) or 600 mg twice daily (with potent inducers) and it is contraindicated in severe renal impairment/ESRD on dialysis when given with potent inhibitors [52]. Cost and the twice-daily, tropism-gated requirement, alongside the availability of cheaper generic co-formulated integrase-based regimens, have limited maraviroc’s role [53].

### 3.6. Long-Term Outcomes and Combination Therapy

Routine-care cohorts confirm durability and tolerability. A Utrecht cohort followed 111 heavily pretreated patients for a median maraviroc exposure of 49 months (up to ~10 years) with continued CD4 rises up to 9 years, treatment failure in only 2.7% and intolerance-related discontinuation in 8.1% [54]. The large European survey (1381 patients, 26 cohorts) reported 82.1% one-year retention despite frequent non-standard use [55]. French database and Italian/Spanish cohorts likewise showed high suppression and immune recovery in R5 patients with markedly worse outcomes and higher discontinuation among non-R5 patients [56].

Beyond antiviral activity, maraviroc has been studied for immunomodulation, since CCR5 blockade also antagonizes chemokine ligands (RANTES, MIP-1α/β, MCP-1) [57]. Larger CD4 gains than attributable to viral suppression alone were observed in MERIT, accompanied by earlier declines in CD38 expression and D-dimer [58]. However, randomized intensification trials in immunologic nonresponders (ACTG A5256, van Lelyveld, Rusconi, Hunt) consistently failed to show meaningful CD4 improvement. Some even showed paradoxical increases in T-cell activation, though apoptosis markers decreased [34,59,60]. A recent 2025 study suggested maraviroc added at ART initiation reduces IL-18 and downregulates the chemokine-signaling inflammatory pathway [61]. Maraviroc has also been repurposed for GVHD prophylaxis, reducing acute visceral GVHD via modulation of alloreactive T cells [62,63].

### 3.7. Resistance and Treatment Failure

Two mechanistically distinct failure patterns predominate. Most commonly, failure results from outgrowth of pre-existing CXCR4-using virus undetected at baseline rather than de novo coreceptor switch. In MOTIVATE, CXCR4-using virus was detected at failure in ~55% of maraviroc failures versus ~9% of placebo failures and clonal/phylogenetic analyses showed these variants were pre-existing minority lineages unmasked by selective suppression of R5 virus [64,65]. Deep sequencing detected pretreatment CXCR4 subpopulations in ~70% of non-R5 failures and geno2pheno false-positive rates fell sharply from screening to failure [66]. Reassuringly, post-maraviroc reversion to R5 tropism was common and even patients failing with X4 virus had greater CD4 gains than the placebo [64,65].

Less commonly, true R5-tropic resistance develops through viral adaptation to use the drug-bound CCR5 coreceptor, manifesting phenotypically as reduced maximal percent inhibition (a plateau below 100%) rather than an EC_50_ shift [64]. The responsible V3-loop and gp120 mutations are patient-specific, context-dependent and lack a consistent signature, making them unpredictable [64,67]. Independent predictors of failure in routine care include low-nadir CD4, detectable baseline viral load, prior PI experience, non-R5 tropism and companion-drug resistance; roughly two-thirds of R5 failures in registrational trials also carried background-drug resistance [55]. In PrEP evaluation (HPTN 069/A5305), all seroconversions occurred with absent, low or variable drug concentrations and maraviroc alone was inadequate, thus underscoring that instead of intrinsic resistance, adherence and combination partners drive most real-world failures [68].

### 3.8. CCR5 Modulation by Maraviroc Beyond HIV: Graft-Versus-Host Disease and Cancer

**Figure 3:**
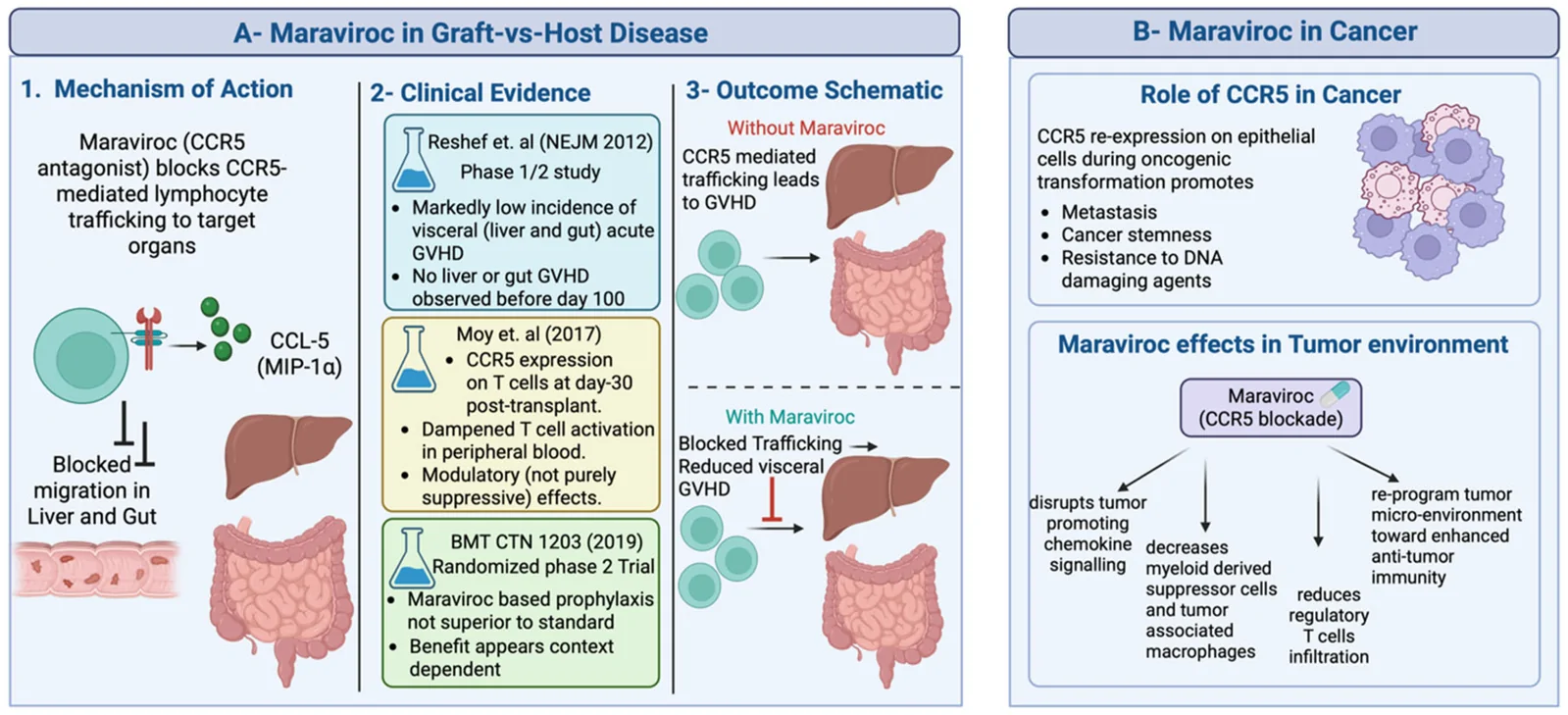
Role of maraviroc in GVHD and cancer [62,70,72].

The regulatory effects of maraviroc on CCR5-mediated lymphocyte trafficking have been exploited therapeutically beyond HIV [69]. The rationale for CCR5 blockade in GVHD derives from the central role of chemokine-directed lymphocyte trafficking in the migration of alloreactive donor T cells to visceral target organs (liver and gut) after allogeneic hematopoietic stem cell transplantation (allo-HSCT), also shown in Figure 3. Maraviroc mimics the functional consequences of the CCR5∆32 genotype, which has itself been associated with reduced grade 3–4 acute GVHD in meta-analysis [69]. The landmark proof of concept came from Reshef et al., who enrolled 38 high-risk patients in a single-arm phase 1/2 study combining maraviroc (300 mg twice daily from day −2 to day +30) with standard tacrolimus/methotrexate prophylaxis after reduced-intensity conditioned allo-HSCT [70]. Maraviroc inhibited CCR5 internalization and lymphocyte chemotaxis in vitro without impairing T-cell effector function or hematopoietic colony formation and patient serum retained antichemotactic activity in vivo [70]. Clinically, the cumulative incidence of grade II–IV acute GVHD was 14.7 ± 6.2% at day 100 and 23.6 ± 7.4% at day 180, with no liver or gut GVHD observed before day 100 and a strikingly low incidence of grade III–IV disease (5.9%) at day 180. These outcomes were favorable given a high-risk population (68% over age 60, 50% matched unrelated and 16% mismatched donors), in whom acute GVHD rates typically exceed 50%, and were achieved without excess relapse, impaired engraftment or increased infection [70]. This represented one of the first demonstrations of a target-organ-specific biologic anti-GVHD effect [71].

Mechanistic follow-up by Moy et al. compared the maraviroc cohort to contemporaneous controls receiving standard prophylaxis alone and confirmed a lower incidence of acute GVHD without increased relapse, together with reduced gut-specific injury markers [62]. Importantly, at day 30 maraviroc increased CCR5 expression on T cells while dampening peripheral T-cell activation and did not impair early immune reconstitution or raise infection risk, hence supporting a modulatory rather than broadly immunosuppressive mechanism. Patients who developed acute GVHD despite maraviroc showed increased T-cell activation, naive T-cell skewing and elevated serum CXCL9/CXCL10, implicating CXCR3 signaling as a potential escape pathway to CCR5 blockade and providing a rationale for combined chemokine-axis targeting [62].

Feasibility was subsequently extended to pediatric and young adult recipients by Khandelwal et al. in a phase II study of 17 patients, in which maraviroc was added to a calcineurin inhibitor-based backbone from day −3 to day +30. No patient developed liver GVHD by day 100 and pharmacodynamic assays confirmed functional CCR5 blockade [63]. However, the trial highlighted a key practical limitation: seven patients discontinued maraviroc early (median day +14), predominantly because of study-defined hepatotoxicity rules, which constrained interpretation of efficacy and underscored the challenge of overlapping hepatic toxicity with transplant conditioning and concomitant agents [63].

The most rigorous test came from the randomized phase 2 BMT CTN 1203 trial, which compared three experimental prophylaxis regimens (including tacrolimus/methotrexate/maraviroc) against a non-randomized contemporaneous control of tacrolimus/methotrexate after reduced-intensity conditioning [72]. Using a composite endpoint (severe acute GVHD, chronic GVHD requiring systemic immunosuppression, relapse or death), post-transplant cyclophosphamide was the most promising arm, whereas the maraviroc and bortezomib arms produced outcomes similar to the methotrexate/calcineurin-inhibitor control [72]. Thus, despite compelling early single-arm and mechanistic data, maraviroc did not demonstrate superiority in the randomized setting, indicating that any clinical benefit is likely context-dependent (influenced by conditioning intensity, donor type, dosing duration and adherence) and that CCR5 blockade alone may be insufficient given compensatory CXCR3-driven trafficking [62,72].

### 3.9. Maraviroc in Cancer

In oncology, the CCL5/CCR5 axis is co-opted by tumors to support both cell-intrinsic malignant behavior and an immunosuppressive tumor microenvironment (TME) [73,74], also shown in Figure 3. Whereas CCR5 is normally restricted to immune cells, oncogenic transformation induces its aberrant re-expression on epithelial tumor cells, where it activates PI3K/Akt, JAK/STAT3, MAPK/ERK and NF-κB signaling to drive proliferation, extracellular matrix remodeling, migration, the epithelial–mesenchymal transition (EMT), angiogenesis and metabolic reprogramming [74,75]. Jiao et al. showed that CCR5+ breast cancer epithelial cells form mammospheres and initiate tumors with more than 60-fold-greater efficiency and that reintroduction of CCR5 promotes metastasis and upregulates DNA-damage repair gene expression and activity [76]. Consequently, CCR5 antagonists (maraviroc, vicriviroc) markedly enhance killing by DNA-damaging chemotherapy, offering a rationale for combining CCR5 blockade with cytotoxic or radiation therapy and potentially permitting dose reduction [73,76].

On the immunologic side, tumor- and stroma-derived CCL5 recruits regulatory T cells (Tregs), myeloid-derived suppressor cells (MDSCs) and tumor-associated macrophages (TAMs); polarizes macrophages toward an M2 phenotype; and stabilizes PD-L1. This collectively establishes an immune-excluded, checkpoint-resistant TME [77,78]. Ban et al. demonstrated that an autocrine CCL5–CCR5 axis is a master regulator of immunosuppressive myeloid cells; its disruption abrogated granulocytic MDSC and TAM generation, enhanced intratumoral neutrophil/macrophage maturation, increased cytotoxic CD8+ T-cell infiltration and reduced Tregs, with combined nanoparticle CCL5 silencing plus maraviroc producing robust antitumor immunity in triple-negative breast cancer models [79]. Halvorsen et al. similarly showed that maraviroc reduces CCL8-driven migration of CCR5+ Tregs into the lungs and decreases metastatic mammary tumor burden [80]. Notably, the axis is context-dependent: in immune-activating conditions CCL5/CCR5 signaling can also recruit cDC1s, NK cells and effector CD8+ T cells and thereby strengthen antitumor immunity, and elevated CCL5 has been associated with both favorable and unfavorable immunotherapy outcomes across tumor types [77,81]. This duality means CCR5 blockade may be beneficial only in tumors where the suppressive arm predominates [77].

Preclinically, maraviroc exerts direct antineoplastic effects in colorectal cancer-inducing G1 cell-cycle arrest, caspase-dependent apoptosis and reduced proliferation, migration and clonogenicity in vitro and inhibiting colorectal liver metastasis in vivo [82,83]. The strongest support for combination with immunotherapy comes from models showing that CCR5 (or dual CCR2/CCR5) blockade reprograms the myeloid compartment and synergizes with anti-PD-1 therapy and radiation, so reducing M-MDSC, Treg and M2-TAM infiltration while increasing effector and memory T cells in glioma and pancreatic adenocarcinoma [84,85].

Clinical translation is shown by the PICCASSO phase I trial (Haag et al.). In this trial, maraviroc was combined with pembrolizumab to treat patients with refractory mismatch-repair-proficient/microsatellite-stable metastatic colorectal cancer, a type of cancer that usually does not respond well to PD-1 inhibitors [86]. The combination was safe and feasible (feasibility rate 94.7%, only one grade 4 event) and translational analyses showed increased antitumoral chemokines during treatment with eotaxin emerging as an overall-survival-linked biomarker [86]. However, objective activity was limited (ORR 5.3%, median PFS 2.1 months), although prolonged disease stabilization in individual patients and better-than-expected overall survival (median 9.8 months) in a heavily pretreated population were observed [86]. Maraviroc may also help reduce treatment-related toxicity. In a breast cancer mouse model, it reduced doxorubicin-induced neuroinflammation and cognitive impairment by inhibiting the NF-κB/NLRP3 pathway. At the same time, it enhanced the antitumor effect of doxorubicin [87].

The most recent syntheses (2025–2026) reinforce the CCL5/CCR5 axis as a multifaceted but nuanced target, emphasizing rational combination with checkpoint inhibitors, chemotherapy, and radiation and calling for biomarker-driven patient selection through single-cell phenotyping and structural–functional mapping of the axis [77]. Contemporary mechanistic work continues to validate maraviroc-targetable circuits, such as the hepatic stellate cell-derived CCL5/CCR5–PI3K-AKT loop driving breast cancer liver metastasis [88]. Alongside maraviroc, other CCR5-directed agents (vicriviroc and the humanized monoclonal antibody leronlimab) are in clinical development for metastatic solid tumors, broadening the therapeutic repositioning of this axis [73,75].

In summary, maraviroc’s activity beyond HIV illustrates the dual therapeutic logic of CCR5 blockade, i.e., interrupting pathologic lymphocyte trafficking in GVHD and disrupting the protumorigenic CCL5/CCR5 circuit in cancer. In both settings, single-agent CCR5 antagonism has produced encouraging biology and early signals but limited definitive clinical benefit, positioning maraviroc most plausibly as a component of rationally designed combination regimens rather than a standalone therapy.

### 3.10. Maraviroc in the Contemporary Antiretroviral Landscape: Comparison with INSTIs and Newer Agents

Although maraviroc validated CCR5 as a druggable antiretroviral target, its place in routine HIV treatment has been progressively eclipsed by the integrase strand transfer inhibitor (INSTI) class. Understanding this divergence clarifies both why maraviroc is now rarely used as conventional therapy and why its distinctive pharmacology retains value for cure-directed and niche applications.

### 3.11. Displacement by INSTIs in First-Line Therapy

Contemporary treatment guidelines converge on second-generation INSTIs [bictegravir (BIC) and dolutegravir (DTG)] as the anchor agents for initial ART in most people with HIV. The 2026 DHHS panel recommends BIC/tenofovir alafenamide (TAF)/emtricitabine (FTC), DTG plus TAF or tenofovir disoproxil fumarate (TDF) plus FTC or lamivudine (3TC), or coformulated DTG/3TC as preferred regimens (all rated AI), citing demonstrated efficacy, a high barrier to resistance, tolerability, low drug–drug interaction potential and convenience [89,90]. The 2024 IAS-USA panel similarly designates BIC- or DTG-based regimens as preferred for most patients (AIa), emphasizing high suppression rates, infrequent toxicity and low pill burden [89]. This positioning reflects direct structural disadvantages of maraviroc rather than a lack of antiviral activity.

Several features render maraviroc noncompetitive as routine first-line therapy relative to INSTIs:

Mandatory tropism testing: Maraviroc works only against HIV that uses the CCR5 coreceptor. Therefore, a phenotypic or genotypic tropism test is needed before starting treatment. This extra step is not required for integrase inhibitors (INSTIs), which work regardless of the coreceptor used by the virus. As a result, maraviroc is less suitable for rapid ART initiation. In contrast, BIC- and DTG-based regimens can be started before resistance test results are available because they have a high barrier to resistance [90].

Lower barrier to resistance and companion-drug dependence: Real-world maraviroc failure is driven largely by unmasking of pre-existing CXCR4-using virus and by companion-drug resistance, unlike the rarity of treatment-emergent resistance to BIC or DTG.

### 3.12. Comparative Efficacy and Tolerability Data

Head-to-head randomized data directly comparing maraviroc with second-generation INSTIs in initial therapy are lacking, reflecting maraviroc’s marginalization before the DTG/BIC era. Indirect evidence nonetheless favors INSTIs [91]. A network meta-analysis of treatment-naive patients found that DTG is one of the most effective core HIV treatments up to 96 weeks. DTG provided better viral suppression than boosted protease inhibitors, efavirenz and cobicistat-boosted elvitegravir. It also showed slightly better suppression than raltegravir and bictegravir. These benefits were especially clear in patients with a high baseline viral load (>100,000 copies/mL) or low CD4 counts (≤200 cells/µL). These are also the patients in whom maraviroc is more limited because the virus may shift to using CXCR4 [91]. Between the two preferred INSTIs, BIC/TAF/FTC was noninferior to DTG-based regimens in the GS-US-380-1489/1490 phase 3 trials, with no treatment-emergent resistance, and in the 1490 study, where there were fewer drug-related adverse events with bictegravir [92]. Maraviroc’s own pivotal efficacy established in the treatment-experienced MOTIVATE trials and the treatment-naive MERIT trial (where it narrowly missed the <50 copies/mL endpoint against efavirenz before tropism-assay refinement) was benchmarked against efavirenz, an agent now itself relegated to alternative status because of central nervous system and rash toxicity relative to INSTIs [53].

Maraviroc’s tolerability profile is favorable in absolute terms (placebo-like adverse-event rates, fewer discontinuations than efavirenz), but this does not translate into a competitive advantage against INSTIs, which combine comparable tolerability with once-daily dosing and no tropism prerequisite. A frequently cited theoretical benefit, i.e., immunomodulation and enhanced CD4 recovery through CCR5 blockade, was not confirmed in randomized intensification trials of immunologic nonresponders, further weakening the case for maraviroc over standard INSTI therapy in this niche.

### 3.13. Maraviroc in the Context of CCR5 Gene-Editing Cure Strategies

The success of CCR5-Δ32 homozygous stem cell transplantation in curing HIV in the “Berlin” and “London” patients has catalyzed gene-editing approaches using CRISPR/Cas9 and zinc finger nucleases to disrupt CCR5 [1]. Maraviroc occupies a complementary niche in this landscape. While gene editing aims for permanent CCR5 ablation, maraviroc provides reversible, pharmacological CCR5 blockade that can be combined with gene-editing strategies or used as a bridge therapy [93]. Recent reviews emphasize the importance of integrating CCR5 expression regulation at transcriptional and post-transcriptional levels with traditional pharmacological approaches like maraviroc to develop more effective combination strategies toward a functional HIV cure [93].

### 3.14. Integrating Maraviroc with CCR5 Gene Editing in Future Cure Paradigms

The “gene–immune synergy” concept positions maraviroc and CCR5 gene editing as complementary rather than competing, an integrated framework increasingly endorsed in recent reviews. Maraviroc could serve as a bridge around the engraftment window of CCR5-edited cells, pharmacologically shielding unedited cells from R5-tropic infection and offsetting the low (~5–14%) editing efficiencies seen in first-in-human trials [1]. Because edited CD4+ T-cell infusion has yielded post-rebound viral control and reinvigorated HIV-specific immunity, pairing maraviroc’s latency-reversing and reservoir-lowering activity (the “kick”) with edited-cell effector function (the “kill”) could operationalize a genuine gene-therapy/shock-and-kill hybrid [94]. Key pitfalls include shared vulnerability to CXCR4-tropic switching—mandating multiplex editing—plus compensatory chemokine escape, off-target mutagenesis, loss of maraviroc’s reversibility, and prohibitive cost [89]. Gene-immune synergy is therefore best framed as a personalized, tropism-stratified strategy for R5-confirmed, transplant-eligible, or heavily treatment-experienced patients, contingent on resolving tropism switching, editing efficiency, and economic feasibility [1].

### 3.15. A Critical Comparison of Gene Editing Versus Pharmacological CCR5 Inhibition

Gene editing (CRISPR/Cas9, ZFN) offers a potentially one-time, durable functional cure by permanently ablating CCR5, but is limited by low editing efficiency, off-target risk, delivery and manufacturing costs, no protection against X4-tropic switching, and residual susceptibilities (e.g., West Nile virus)—confining it to research and heavily treatment-experienced or transplant-eligible patients [1,94,95]. Pharmacological inhibition (maraviroc) provides reversible, titratable, well-tolerated blockade but requires lifelong twice-daily dosing and exclusively R5 tropism, best suiting virologically suppressed R5 patients needing salvage or immunomodulation [48,93]. The approaches are complementary, with maraviroc serving as a bridge or adjunct within editing-based cure strategies [93].

### 3.16. A Forward-Looking Perspective for CCR5-Targeted HIV Therapeutics

A promising future approach is to use in vivo-based editing of CCR5 in HPSCS. Helper-dependent adenoviral vectors have achieved about 50% CCR5 editing and provided long-lasting resistance to HIV without detected off-target effects. This approach could be a more efficient and affordable alternative to ex vivo stem cell transplantation [7]. Multiplex and combinatorial editing (CCR5 plus CXCR4/LTR or C46 fusion inhibitors) addressing tropism switching and maraviroc’s repositioning in oncology and GVHD are also advancing [8,77]. Major unanswered questions should guide future research: How can editing efficiency and delivery be optimized while minimizing off-target mutagenesis and immune rejection? [1,96]. Can CCR5-only strategies be safeguarded against X4-tropic emergence? [8]. How will durability, long-term safety and global affordability be ensured? [97]. Finally, which patient subgroups (particularly CCR5Δ32 heterozygotes) benefit most and how should gene editing be integrated with latency reversal, broadly neutralizing antibodies and immune-based interventions to achieve a scalable functional cure? [94,96].

## Figures and Tables

**Table 1:** CRISPR/Cas9 strategies targeting CCR5 for HIV therapy.

Sr. No.	CRISPR-Based Strategy	Target/Approach	Model	Key Findings	Major Limitations/Considerations	Reference
1	CCR5 knockout using SpCas9	Disruption of CCR5 through Cas9-mediated DSB and NHEJ	CD34+ HSPCs; humanized mice	CCR5 ablation conferred HIV-1 resistance and edited cells were selectively enriched following HIV challenge	Editing efficiency remains a major barrier to achieving sufficient protection	[19]
2	CCR5 editing using SpCas9	Smaller S. aureus Cas9 targeting CCR5	Primary CD4+ T cells and CD34+ HSPCs; humanized mice	Promoted HIV-1 resistance and selective enrichment of edited CD4+ T cells	Requires careful optimization of delivery and editing efficiency	[17]
3	HDR-mediated CCR5Δ32 recreation	Precise introduction of the naturally protective CCR5Δ32 mutation	iPSCs and differentiated monocytes/macrophages	Biallelic targeting reached up to 33%; differentiated cells demonstrated resistance to HIV-1	HDR is generally less efficient than NHEJ and requires a donor template	[15]
4	Biallelic selectable knock-in	CRISPR/Cas9-mediated HDR with selectable biallelic CCR5 disruption	Human cells	Enabled selection of cells carrying frameshift mutation in both CCR5 alleles and inhibited HIV-1 infection	Additional selection/manufacturing steps may complicate clinical translation	[16]
5	Dual CCR5 + HIV proviral DNA targeting	Simultaneous disruption of host CCR5 and integrated HIV-1 proviral DNA	HIV-infected humanized mice	Eliminated replication-competent virus in 58% of infected animals under ART	Requires efficient delivery to both infected target cells and viral reservoirs	[5]
6	In vivo CCR5 base editing	Base editors delivered using helper-dependent adenoviral vectors	HSCs; humanized mice	Approx 50% of CCR5 editing in bone marrow mononuclear cells and ~12-fold-lower plasma HIV titers	In vivo delivery, durability, immunogenicity and long-term safety remain important challenges	[7]
7	CCR5 knock-out + C46 Inhibitor	CCR5 disruption combined with membrane-anchored HIV fusion inhibitor C46	Cell line models	Provided resistance against both R5 and X4 tropic HIV-1, superior to either strategy alone	Requires combination engineering and validation in clinically relevant models	[4]
8	CCR5 + CXCR4 targeting	Simultaneous disruption of both HIV coreceptors	T cells; humanized mice	Broad resistance against R5, X4 and dual-tropic HIV-1	CXCR4 disruption is problematic in HSPCs because CXCL12/CXCR4 signaling is essential for bone marrow homing and maintenance	[20,21,22,23,24]
9	High-fidelity Cas9	HiFi Cas9 variant delivered as an RNP	Human HSPCs	Maintained efficient CCR5 targeting while reducing off-target editing	Requires further long-term clinical safety validation	[18]

## Data Availability

No new data were created or analyzed in this study. Data sharing is not applicable to this article.
